# Content Recommendation Systems in Web-Based Mental Health Care: Real-world Application and Formative Evaluation

**DOI:** 10.2196/38831

**Published:** 2023-01-19

**Authors:** Akhil Chaturvedi, Brandon Aylward, Setu Shah, Grant Graziani, Joan Zhang, Bobby Manuel, Emmanuel Telewa, Stefan Froelich, Olalekan Baruwa, Prathamesh Param Kulkarni, Watson Ξ, Sarah Kunkle

**Affiliations:** 1 Headspace Health San Francisco, CA United States

**Keywords:** behavioral coaching, recommender systems, mental health, machine learning, natural language processing, telemental health, mobile health, mHealth, digital health, digital coaching, health platform

## Abstract

**Background:**

Recommender systems have great potential in mental health care to personalize self-guided content for patients, allowing them to supplement their mental health treatment in a scalable way.

**Objective:**

In this paper, we describe and evaluate 2 knowledge-based content recommendation systems as parts of Ginger, an on-demand mental health platform, to bolster engagement in self-guided mental health content.

**Methods:**

We developed two algorithms to provide content recommendations in the Ginger mental health smartphone app: (1) one that uses users' responses to app onboarding questions to recommend content cards and (2) one that uses the semantic similarity between the transcript of a coaching conversation and the description of content cards to make recommendations after every session. As a measure of success for these recommendation algorithms, we examined the relevance of content cards to users’ conversations with their coach and completion rates of selected content within the app measured over 14,018 users.

**Results:**

In a real-world setting, content consumed in the recommendations section (or “Explore” in the app) had the highest completion rates (3353/7871, 42.6%) compared to other sections of the app, which had an average completion rate of 37.35% (21,982/58,614; *P*<.001). Within the app’s recommendations section, conversation-based content recommendations had 11.4% (1108/2364) higher completion rates per card than onboarding response-based recommendations (1712/4067; *P*=.003) and 26.1% higher than random recommendations (534/1440; *P*=.005). Studied via subject matter experts’ annotations, conversation-based recommendations had a 16.1% higher relevance rate for the top 5 recommended cards, averaged across sessions of varying lengths, compared to a random control (110 conversational sessions). Finally, it was observed that both age and gender variables were sensitive to different recommendation methods, with responsiveness to personalized recommendations being higher if the users were older than 35 years or identified as male.

**Conclusions:**

Recommender systems can help scale and supplement digital mental health care with personalized content and self-care recommendations. Onboarding-based recommendations are ideal for “cold starting” the process of recommending content for new users and users that tend to use the app just for content but not for therapy or coaching. The conversation-based recommendation algorithm allows for dynamic recommendations based on information gathered during coaching sessions, which is a critical capability, given the changing nature of mental health needs during treatment. The proposed algorithms are just one step toward the direction of outcome-driven personalization in mental health. Our future work will involve a robust causal evaluation of these algorithms using randomized controlled trials, along with consumer feedback–driven improvement of these algorithms, to drive better clinical outcomes.

## Introduction

A recommender system (RS) is an algorithm that filters information, content, or decisions into a relevant subset of choices for an individual, using factors such as the user’s usage history and preferences [1]. In addition to their use in social media platforms, these algorithms have become an essential part of modern health care and are used to provide clinical decision support for providers and health care education and behavior change for patients [2-7]. As technology has enabled shifting health care from in-person treatment centers to remote care, RSs can provide tailored web-based care for a variety of purposes, including digital mental health treatment [8].

Digital mental health platforms, ranging from direct-to-consumer apps to telemental health platforms, can increase access to care and help meet the massive demand for mental health services [9,10]. These platforms often offer a host of self-guided psychoeducational content, behavioral exercises, and homework activities that can be therapeutically beneficial to an individual living with specific health conditions and assist with general mental health and well-being. Self-guided content that uses RS can serve as a scalable approach to supplement patients’ mental health journeys.For example, offline access to cognitive behavioral therapy content can help users better understand the techniques and how to practice them. Common applications of self-guided content can be found in meditation apps like Calm and Headspace, which have been shown to reduce stress, improve mental health, and reduce fatigue and pain in numerous populations [11-16].

While engagement with self-guided content is beneficial, there is a need to personalize the content given the multifaceted nature of mental health (eg, patient condition, environment, and psychosocial stressors) [9]. Personalization can help to reduce choice overload, increase digital therapeutic alliance by providing recommendations that increase the likelihood the user feels understood, and support users to self-manage their mental health and well-being [17]. Further, a bolstered therapeutic alliance using RSs can also increase the likelihood of clinical improvement [17].

In this study, we present 2 modalities for delivering these recommendations, onboarding-based and coaching conversation–based content recommendation algorithms, which were deployed in a real-world setting and evaluated with 14,018 users of the Ginger behavioral health coaching and therapy app. These two algorithms deliver recommendations within the same section of the app depending on different user states. Onboarding-based recommendations are used to initiate the care process of consuming content for new users and those that tend to use the app just for content but not for coaching. Conversation-based recommendations update to match the semantic content that users discuss with their coaches. As a formative evaluation, we measure and report the content completion rates of both approaches in a real-world setting. In addition, for the conversation-based content recommendation algorithm, we measure the relevance of recommended content cards to a user’s conversations with their coach through offline expert annotations. Our evaluation supports product and design decisions for content placements but does not allow for causal inference due to a few potential confounders. To the best of our knowledge, this is the first large-scale study to evaluate the effectiveness of mental health content recommendation systems in a real-world setting where patients are being supported with this content. Consequently, we hope that this study will help inform the burgeoning implementation of future digital health RSs across industry and academia.

## Methods

### Participants

This is a retrospective observational study of 14,018 individuals aged 18 years or older who use Ginger, an on-demand mental health app. The users had access to the Ginger app through their employer or health plan benefits. We only included users who used the self-guided content library in the app. Data presented here were collected from the usage patterns of these Ginger users between June and September 2021. We chose this period because it reflects the approximate timing of when all 3 conversation-based recommendation algorithms (explained in detail in the subsequent section) in consideration were serving content recommendations to Ginger users. 

Age and gender demographic data were unreported for 24.8% (n=3476) and 34.5% (n=4836) of users, respectively. Of the individuals that reported age information, 7.19% (n=758) were aged 18 to 24 years, 45.25% (n=4770) were aged 25 to 34 years, 26.07% (n=2748) were aged 35 to 44 years, 20.16% (n=2125) were aged 45 to 64 years, and 1.33% (n=140) were 65 years or older. For users that reported gender, 28.08% (n=2578) were male, 61.63% (n=5659) were female, and 10.2% (n=936) were nonbinary. 

### Ethical Considerations

This study represents a secondary analysis of preexisting deidentified data. The study team does not have access to the participants or their identifying information and does not intend to recontact participants. Ginger’s research protocols and supporting policies were reviewed and approved by Advarra’s institutional review board (Number Pro00046797) in accordance with the US Department of Health and Human Services regulations at Title 45 Code of Federal Regulations Part 46. This study protocol was reviewed by the Advarra institutional review board (IRB) and determined to be exempt from IRB oversight, as deidentified secondary data analysis is generally not regarded as research with human participants.

### Ginger App Content System

Ginger provides web-based on-demand mental health services, primarily through employee or health plan benefits. Using a mobile app platform, Ginger users can access text-based behavioral health coaching, teletherapy, and telepsychiatry, as well as self-guided content and assessments. For self-guided content, users have access to more than 200 clinically validated content cards. These content cards contain curated activities ranging from mindfulness exercises to psychotherapeutic education. The content is presented in a variety of formats, including meditations, breathing exercises, videos, podcasts, surveys, and readings that typically take between 2 and 10 minutes to complete. The Ginger app uses the Amplitude analytics platform to record content-related events emitted by users while using the app [18].

### Content Modalities

**Figure 1:**
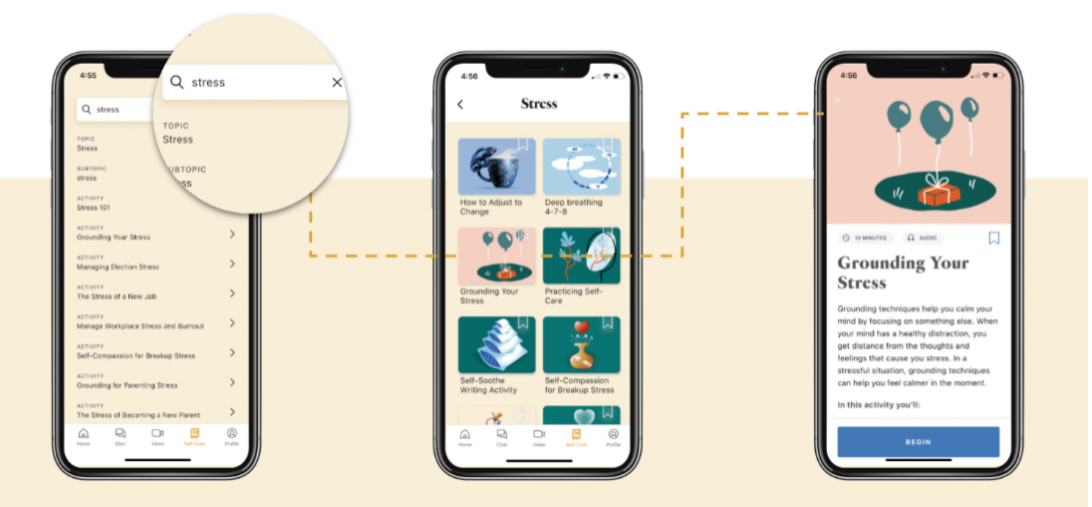
Content search in the Ginger self-care library. Members can access content from several sources in the app, including the Explore section, the content library, and the content search bar. This figure shows how users can access content via the search bar.

**Figure 2:**
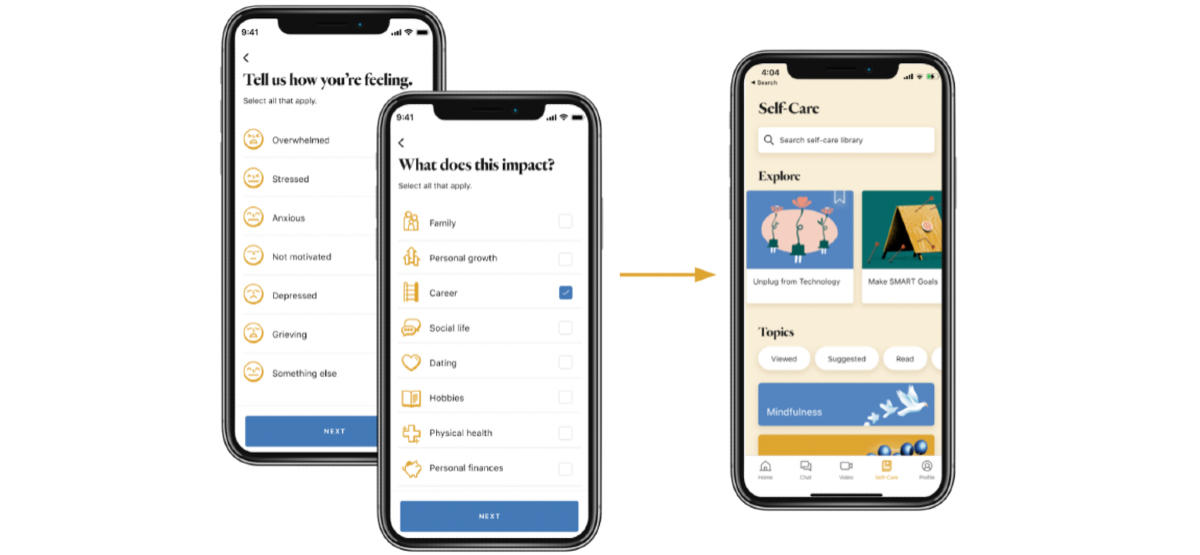
Onboarding response-based recommendations. This figure shows how answering the two onboarding questions can recommend content in the Explore section of the app.

Ginger users generally access content via 1 of several pathways in the mobile app, as depicted in Figures 1 and 2.

First, coaches supplement their text-based coaching sessions by assigning and sending links to content cards as homework.

Second, users access content cards by searching on the self-care tab (Figure 1).

Third, Ginger’s content recommendation system surfaces recommendations under the recommendations (called “Explore” in the app) heading on the self-care tab. 

Finally, users can browse through the content library by traversing through different categories (eg, Job Anxiety, Habit Formation, and Behavior Change) and browsing through various activities within them.

### Content Recommendation Systems

Within the aforementioned Explore section, three algorithms serve recommendations: (1) onboarding-based recommendations, whereby content card suggestions are guided using the user’s onboarding responses, which are provided by all users when signing up for the service (Textbox 1); (2) conversation-based content recommendations, whereby content card suggestions are delivered by an algorithm that utilizes the context and content of the user’s conversation with a mental health coach; and (3) random recommendations, whereby random content card suggestions are provided.

For example, a combination of responses by a user could be Anxious, Depressed, Family, Career, and Something else.

**Onboarding questions in the app and their responses.**

**Question 1: Tell us how you’re feeling. (Select all that apply)**

1. Anxious
2. Depressed 
3. Grieving
4. Not motivated 
5. Overwhelmed 
6. Stressed
7. Something else

**Question 2: Which area(s) does this impact your life? (Select all that apply)**

1. Career
2. Dating
3. Family
4. Hobbies
5. Personal finance
6. Personal growth
7. Physical health
8. Social life
9. Something else

### Recommendation Algorithm Decision Flow Delivered in the App Back End

When a user has a conversation with a coach within the past 60 days that has over 15 messages, they receive conversation-based recommendations. If not, the app defaults to onboarding-based recommendations. The algorithm will default to random recommendations if the user has only selected “something else” for both onboarding questions.

### Onboarding-Based Recommendations

#### Process Overview

Onboarding responses are provided by all users upon signing up for the service (Figure 2, Textbox 1). We developed an algorithm that uses users' responses to their onboarding questions to recommend content cards from the Ginger content library. To do this, we first created a mapping of users' responses to 2 coaching onboarding questions (Textbox 1) to content cards based on how relevant the content card was to the given set of onboarding responses. Second, we built an algorithm that outputs an ordered list of most relevant to least relevant content cards for users with a particular answer set. We explain the specific steps of the algorithm in the following subsections.

#### Step 1: Creating Numerical Mappings of Relevant Answers to Activity Cards 

The ground truth relevance of the content cards to onboarding response labels was gathered through expert annotations. Six certified mental health coaches annotated 170 Ginger content cards [19]. Annotators were instructed to map all possible sets of onboarding responses (eg, depression, anxiety, etc) that the content of a card could help address. The aim of these annotations was to obtain a diverse set of responses for each content card over all annotators that were then aggregated to obtain a normalized label set for each content card. This delivered a mapping between each content card and each onboarding response (eg, 3-minute meditation C_i_: [Anxious: 0.72, Depressed: 0.11, Grieving: 0.02, Social life: 0.2]).

When matching these cards for a user, we constructed a similar vector for the user’s response to onboarding answers (eg, user response U: [Anxious: 1, Depressed: 1, Grieving: 0, Social life: 0]).

#### Step 2: Retrieving Relevant Activity Cards for a User Using Answer-Card Mappings

As we previously mentioned, we obtained the set of derived card mappings and user responses as vectors. Using these vectors, for each user, we computed the cosine similarity of their user onboarding response vector U with each of the content card vectors (C_1_…C_i_…C_n_) [20], which returned an ordered list of content cards from highest to lowest relevance to the user response. Finally, to increase the diversity of recommended cards, we introduced category-based sampling. Each content card has a category associated with it, such as Relaxation, Depression, and Meditations. To perform category-based sampling, we updated the ordered list of activity cards by replacing one-third of the activity cards chosen randomly from the list of recommendations with other cards chosen randomly from the original card’s category. We serve this updated list of activity cards to the product application programming interface.

### Conversation-Based Recommendations

#### Process Overview

**Figure 3:**
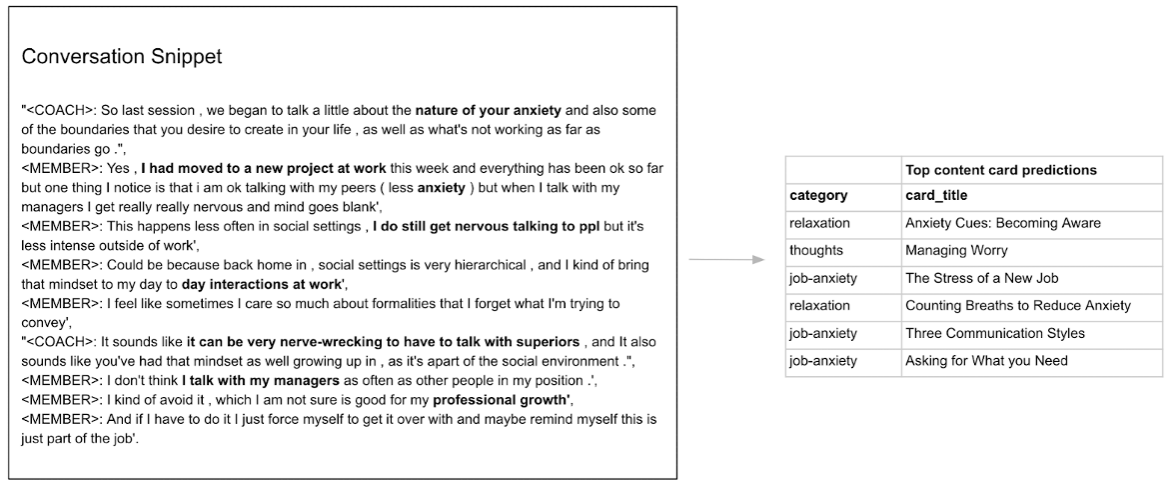
Example output of conversation-based recommendations: User mentions having anxiety due to communication at the workplace. The conversation snippet and corresponding activity card suggestions by the algorithm are shown.

The conversation-based recommendation algorithm works by matching the semantic similarity between the content of a conversation to the text description of content cards to make recommendations suitable for a conversational snippet. An example of a recommendation made by this algorithm for a coach-user conversation is shown in Figure 3. There are 2 main steps involved in recommending content cards based on the coaching session text conversation, which are further illustrated in the following subsections. 

#### Step 1: Filtering Conversation Transcript Using Message Importance

A subset of important messages was extracted from a coach-user conversation and used by the conversation-based recommendation algorithm (Multimedia Appendix 1). This step was crucial to reduce false positives in recommending content cards (ie, ignore messages that may have high overlap with content card text but low levels of importance in a session). 

#### Step 2: Generating a List of Recommendations Using the Filtered Conversation

**Figure 4:**
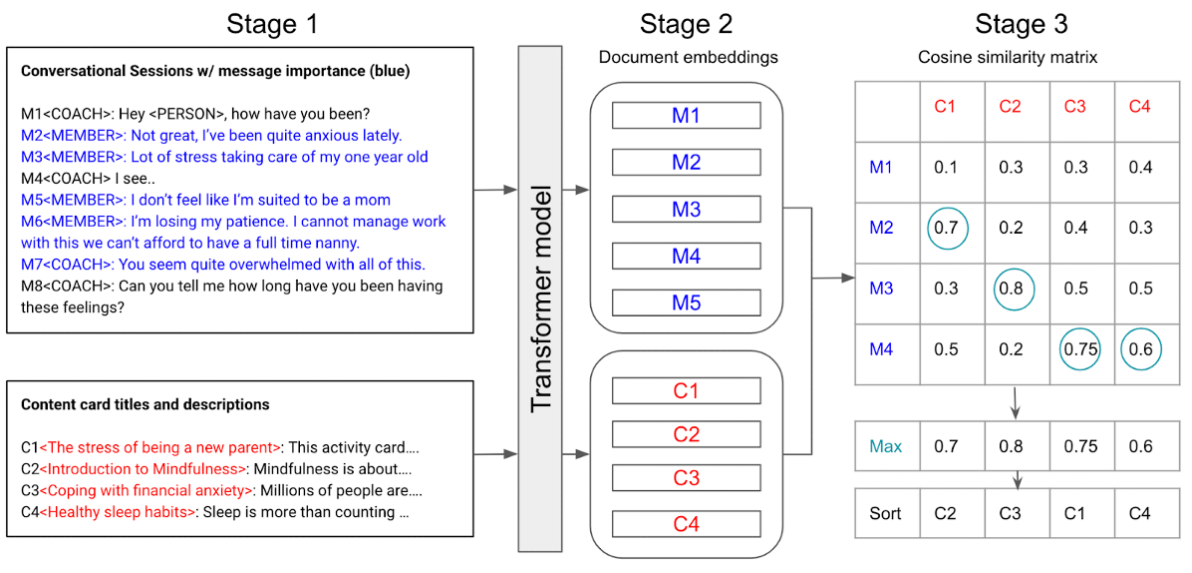
Conversation-based content recommendations. This algorithm provides inference over 3 stages. Stage 1: We algorithmically identify the most important messages in a conversation (text in blue). Stage 2: We mathematically represent the text for sessions and content card description using a natural language model as document embeddings or vectors. Stage 3: For each conversational snippet, we find content cards that are most similar to important messages in the conversation and retrieve the cards with the highest similarity to the text.

The conversation-based recommendation algorithm provided an ordered list of content cards from highest to lowest similarity to the conversational text using an unsupervised method that requires no training data. To do this, both the conversational text and content card descriptions were mathematically represented as embedding vectors [21] using a language model that generated embeddings from text [22]. This model is henceforth referred to as the embedding model. We used a multilingual transformer based on the XLM-Roberta (Fraunhofer Society) architecture [22] pretrained on a paraphrase similarity task [23] as the embedding model [24]. We chose to use a model that is trained to detect paraphrases to identify semantic overlap in the text between the conversational session and the content card descriptions. This particular model was chosen because (1) it is pretrained and did not need any additional training data to initialize the model for inference; (2) it is multilingual, which is key, given that Ginger already serves users in 2 languages (English and Spanish); and (3) XLM-Roberta has state-of-the-art performance for paraphrase similarity task [22]. To generate the embedding vectors from the text, we took the mean of the output layer from the final hidden layer of the embedding model’s neural network. This gave us the text embedding for the sequence under consideration, either the description of a content card or a message in the conversation transcript. To get the final content recommendations from the embeddings shown in Figure 4, we first computed the cosine similarity matrix between all the message embeddings (M_1_…M_i_…M_n_) and all the content card embeddings (C_1_…C_i_…C_n_), with messages representing the rows and cards reflecting the columns of the matrix (Figure 4). Then, we applied a Max Operator function over all columns of the matrix to get the relative relevance of content cards. This gave us the cosine similarity score over the message that was most similar to a content card, thus imparting a stronger emphasis on individual messages within the session text. Finally, we sorted content cards based on maximum similarity scores to get the final ordered list of recommendations.

### Evaluation

#### Evaluation Overview

We evaluated performance both offline and in the app. The offline evaluation informed algorithm design decisions, and the in-app evaluation measured the algorithm’s performance in the real-world setting. For the offline evaluation, we compared the relevance of the conversation-based recommendations to random recommendations. For the in-app evaluation, we compared the completion rates of cards recommended by the conversation-based recommendation algorithm with both the onboarding-based and random recommendations.

#### Offline Evaluation

For the offline evaluation, we computed the probability of a recommended card being relevant (also defined as relevance rate) for the top 5 conversation-based recommendations per conversation and compared it to random recommendations to assess the relative performance of the two algorithms. 

To do this, we bucketed conversation sessions by the number of text messages and reported relevance rates per bucket to understand how relevance varies with the number of messages in a session. The data set for these conversational sessions and recommendation pairs was created by generating batch predictions for 110 randomly selected text sessions between a Ginger coach and a user. The bucketed distributions by the number of messages in the session are shown in Table 1.

The 110 coach-user conversational sessions were annotated using the open-source Doccano annotation tool [25] by 3 licensed mental health coaches. We used 20 sessions overlapping across all 3 coaches to compute the interannotator agreement. For a given conversational session, annotators marked a recommended card as either “somewhat relevant,” “very relevant,” or “not relevant” to the session. To simplify this analysis, cards labeled as “somewhat relevant” or “very relevant” were combined as “relevant.” 

We used the majority agreement rate (MAR) as our metric for interannotator agreement. In a nutshell, MAR calculates how often each annotator agrees with the majority vote from all annotators according to a classification metric such as accuracy, precision, or *F_1_*. Therefore, MAR tells us, on average, how well each annotator predicts the majority. Using MAR with macroaverage *F_1_*, our annotators showed an overall agreement of 84.9%, which is considered medium to high depending on the task. Table 2 lists the MARs for each annotator (using macroaverage *F_1_*).

**Table 1:** Distribution of messages in conversational sessions.

Bucket (messages in session), n	Sessions, %
0-5	28.26
5-10	24.76
10-20	22.07
20-40	14.13
40 and above	10.76

**Table 2:** Interannotator agreement.

Answers	Annotator 1	Annotator 2	Annotator 3	All
“Not relevant”	0.607143	1.000000	1.000000	0.869048
“Relevant”	1.000000	0.621622	0.864865	0.828829
Macroaverage	0.803571	0.810811	0.932432	0.848938

#### In-App Evaluation

We measured content card completion rates in the app’s Explore section for all 3 served algorithms (ie, random recommendations, onboarding-based recommendations, and conversation-based recommendations) spanning 68 days between June 2021 and September 2021 that served recommendations to 14,018 users. We also measured the completion rate of cards via the recommendations section compared to other sections of the app where cards are not recommended by these algorithms. These sections included the Content Library, Home Screen, Search, and coaching recommendations through chat. Additionally, to understand the effects of age and gender on content completion, we measured and compared completion rates of content consumed via different algorithms in the recommendation section by stratifying users by age and gender. The card completion rate is the ratio of the number of times content cards were completed in a section to the number of times cards were viewed in that respective section of the app in the same time frame. We chose to use the completion rate as a proxy for engagement compared to metrics such as click-through rate since the completion of a content card is more closely tied to a user finishing the desired activity.

## Results

### Offline Evaluation

**Figure 5:**
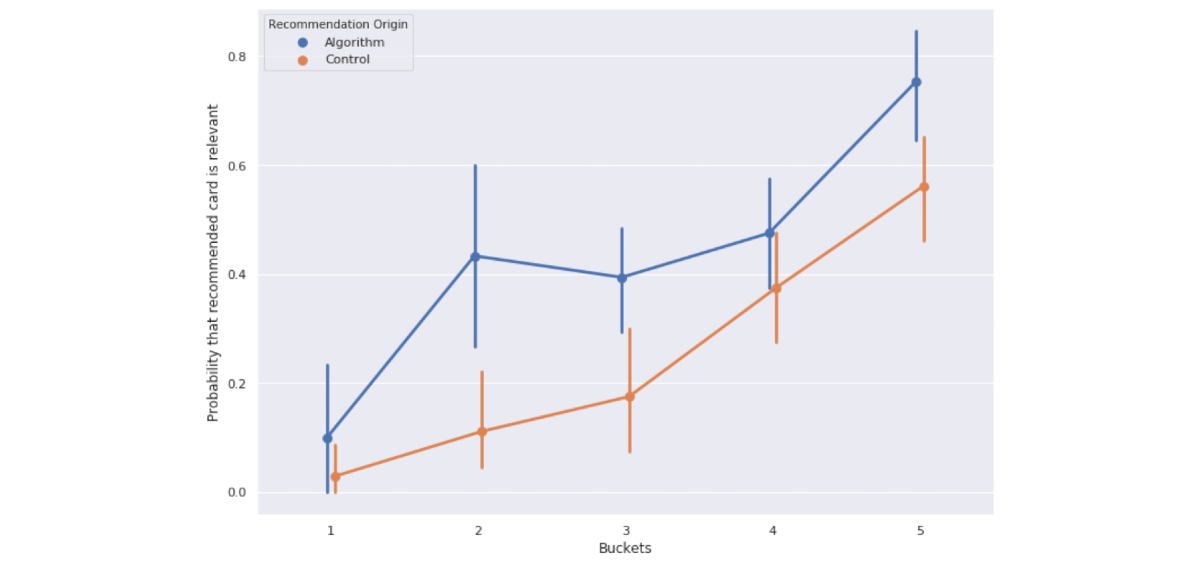
Probability of a recommended card being relevant (relevance rate): conversation-based recommendation measured against the random recommendation control.

We compared the relevance rates between the conversation-based recommendations and the random control recommendations using a paired *t* test for each session length category. As enumerated in Table 3 and illustrated in Figure 5, the conversation-based algorithm had a higher relevance rate across all categories: 0 to 5 messages (*P*=.23), 5 to 10 messages (*P*=.001), 10 to 20 messages (*P*=.12), 20 to 40 messages (*P*=.20), and 40 or more messages (*P*=.01). The random recommendations’ performance reflects the baseline relevance of all content cards in the library to the conversational sessions. The difference between the computed probability of recommending a relevant card (relevance rate) in the top 5 random recommendations versus the conversation-based recommendations quantifies the impact of the conversation-based algorithm in providing relevant suggestions. Of note, the relevance rate of the random recommendations is greater than 0 since the content library consists of cards related to mental health, and some of these cards will inevitably be relevant to a mental health coaching conversation. Finally, we observed a trend of increasing relevance as the number of messages increased in a session, both for conversation-based recommendations and random recommendations. 

To test whether session length categories are jointly significant in predicting a differential impact of the random and conversation-based recommendations on relevance rates, we conducted an omnibus test for a model relying on the interaction of session length and recommendation method in predicting relevance rates. The resulting statistic was *F_9,783_*=16.05, with *P*<.001. This omnibus test, which corrects for multiple comparisons, had a Sidak-adjusted *P* of <.001. This supports the model that session length predicts differences in relevance rates by recommendation type. For the individual *t* tests that determine whether the relevance rates differ by recommendation types within session length categories, the corresponding Sidak-adjusted *P* values were as follows: 0 to 5 messages (*P*=.98), 5 to 10 messages (*P*=.46), 10 to 20 messages (*P*=.91), 20 to 40 messages (*P*=.95), and 40 and more messages (*P*=.96). Note that this adjustment reduces the power of the test, which might cause it to incorrectly fail to reject the null hypothesis.

**Table 3:** Performance of the conversation-based algorithm in the offline analysis.

Messages in session, n	Relevance rate for conversation-based recommendation algorithm	Relevance rate for random control	Difference: (algorithm−control)	*P* value	Sidak-adjusted *P* value
0-5	0.099	0.028	0.071	.23	.98
5-10	0.433	0.111	0.322	.001	.46
10-20	0.393	0.175	0.218	.01	.91
20-40	0.47	0.375	0.95	.20	.95
40 and above	0.753	0.561	0.192	.01	.96

### In-App Evaluation 

As shown in Table 4, content cards from the Recommendations/Explore section in the app had the highest completion rates (3353/7871, 43%) across all sections of the app, followed by content on the home screen (8679/21,863, 40%) and recommendations made in coaching sessions (2698/7313, 37%), with *P*<.001 when comparing the recommendation section to all other content serving sections of the app. Conversation-based recommendations (1108/2364) had 11.4% (1712/4067; *P*=.003) higher completion rates per card than onboarding response-based recommendations and 26.1% (534/1440; *P*=.005) higher than random recommendations.

**Table 4:** Click and completion rates across content sources.

Content source	Clicks, n	Completions, n	Completion rate, %	Precision rate (K=5), %
Home page	21,863	8679	0.397	N/A^a^
Library	20,291	6939	0.342	N/A
Recommendations	7871	3353	0.426	N/A
Conversations	2364	1108	0.469	0.169
Onboarding responses	4067	1712	0.421	0.149
Random	1440	534	0.371	0.165
Coach chat	7313	2698	0.369	N/A
Other	7707	2974	0.386	N/A

^a^N/A: not applicable.

These *P* values were computed using 2-sided *t* tests between the distributions of mean content card completion grouped by users for different sections of the app (eg, the recommendations section versus all other sections of the app) and different recommendation algorithms (eg, the conversation-based content recommendation method versus onboarding recommendations).

**Figure 6:**
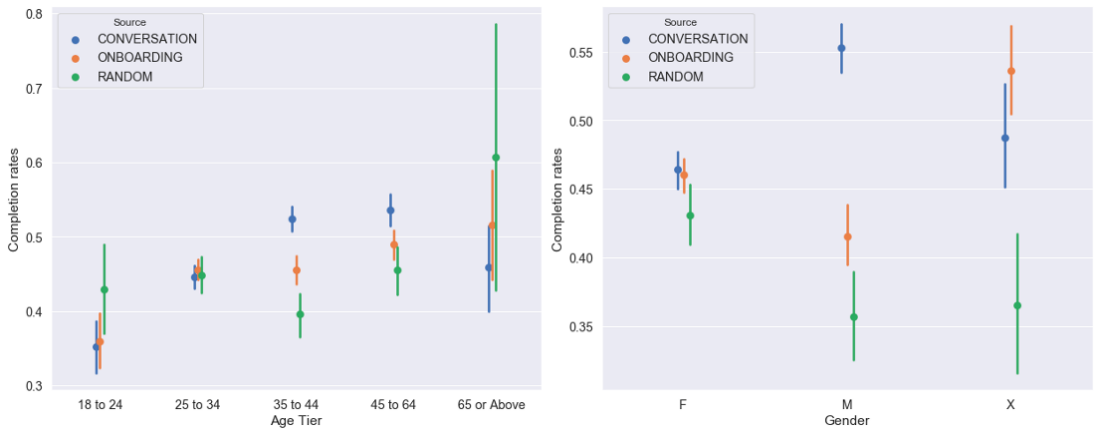
Completion rates of content delivered via the three different algorithms across different age and gender categories. Note that this point plot was plotted with the Seaborn Python library using a bootstrapped sampling of data points to generate confidence intervals.

To observe if certain groups of age or gender demographics were less or more receptive to personalized recommendations, we created point plots splitting the completion rates of content delivered via the three different algorithms by age and gender categories (Figure 6). We observed that both age and gender variables were sensitive to different recommendation methods. A user was more likely to respond to conversation-based recommendations if they were aged 35 years and up. Additionally, we noticed that our male-identifying population had a higher propensity to respond to conversation-based recommendations compared to our female-identifying population. For the nonbinary population, it is hard to make claims since the total population size for this study was small, resulting in much larger confidence intervals.

To test whether demographics jointly predict differential completion rates by recommendation type, we conducted *F* tests for the model predicting completion rates using either the interaction of age bins with recommendation types or the interaction of gender with recommendation types. The *F* statistic corresponding to the model using gender was F_2,20454_=4.39 (*P*=.01), and that corresponding to the model using age was F_5,33226_=21.43 (*P*<.001). The degrees of freedom differed for these 2 models due to the different number of users with nonmissing age and gender information. These results indicate that different groups of age and gender tend to respond differentially to different degrees of personalization.

## Discussion

### Principal Findings

In this study, we presented 2 personalized methods for delivering content recommendations, namely the onboarding-based and conversation-based content recommendation algorithms. As a measure of the impact of recommendations, we observed that the recommendations section had overall higher completion rates compared to the content in other sections of the app. For the different algorithms used in this study, we noticed that the conversation-based content recommendations had the highest completion rates in the Explore section of the app over onboarding-based recommendations and random recommendations. Finally, we saw that both age and gender variables were sensitive to different recommendation methods with responsiveness to conversation-based recommendations being higher if the users were 35 years or older or identified as male.

### Additional Findings

#### Recommended Content Has Higher Engagement Compared to Content in Other Sections of the App

Completion rates of content activity cards in the Explore (recommendations) section versus other sections of the app, including browsing the content library and content embedded in the chat conversations, were higher, with a 42.6% (3353/7871) completion rate. This points to the higher engagement of users in these sections. One possible confounding factor for this observation could be that the recommendations shelf lives on top of the self-care tab (Figure 2, right) and is thus more accessible than the default content card library (lower on the same page). However, the home screen and the chat tab require fewer clicks for access but have lower completion rates than the recommendations, suggesting that this confounding factor's impact is minimal. An interesting observation was that coach chat–recommended content given as homework had lower completion rates than the Explore section. While we have not identified the exact reasons for this, we believe that scrolling back on multiple messages after completing a session with a coach might be more cumbersome than browsing through the self-care content tab. These findings highlight the need to consider design thinking principles (eg, content placement) when using apps to deliver content to users. 

#### Conversation-Based Recommendations Have Higher Engagement Than Onboarding-Based and Random Recommendations

All 3 recommendation algorithms live in the same section of the app, so they could be compared without the effect of placement in the app. Conversation-based recommendations had the highest completion per card compared to onboarding-based recommendations and random recommendations. The increased relevance of content cards is associated with increased user engagement and content card completion. We purport that onboarding-based recommendations outperformed random recommendations because they were personalized to the user’s onboarding answers. Similarly, conversation-based recommendations had higher engagement rates than onboarding-based and random recommendations. We hypothesize that this was because conversation-based recommendations dynamically update as a user chats with their coach, facilitating a better care experience across the app. 

#### Longer Conversational Session Lengths Drive More Relevant Content Recommendations

During the offline analysis, we observed a trend of increasing relevance as the number of messages increased in a session. This is primarily an artifact of the algorithm design since there is a higher chance that a longer conversational session will recommend more relevant content when more topics are discussed. However, this result motivated our decision to establish a threshold of 15 messages (or an average relevance score of ~0.4 for the 10 to 20 message bucket) as the minimum number of messages required for a session to trigger conversation-based recommendations in the Ginger app. 

### Limitations

One limitation of this work is that we cannot derive causal inferences from the results of this study, as content card recommendation completion could be driven by numerous factors besides the recommendation algorithm itself. The three different algorithms were not served randomly across the user population; rather, a user’s baseline level of engagement determined which recommendation system they were served. There might be other confounding factors associated with users attending their coaching sessions (which means they have sessions to use for recommendations) and being more motivated to complete and update their onboarding responses. Additionally, engagement can vary with confounders such as time of day and year, baseline Patient Health Questionnaire and Generalized Anxiety Disorder Assessment scores, and user resilience [26]. For example, the beginning of the year usually sees higher engagement due to a resolution mindset. Additionally, users’ level of baseline anxiety, depression, and resilience can impact their ability to start and complete the content assigned to them, which could further affect our defined engagement metric.

Another limitation of this work is in the choice of our user engagement metric, the card completion rate. While the completion rate is a good proxy for understanding if users are engaging with content that they click on, it does not indicate the attractiveness of a content item. This value is better served by looking at the click-through rate, which is the probability that a user will click on an item after viewing it. Unfortunately, it is difficult to estimate click-through rates in the current version of the Ginger app across different devices of different sizes. For this reason, we chose to only use the completion rates as our main metric of relevance.

Finally, our results indicate differential content completion across demographics with recommendation algorithm type, however the reasons for this occurrence are not known to us at presenty. This will be the focus of a future qualitative study.

### Broader Implications

Our findings suggest that recommended content has better engagement than other sections of the Ginger app. Thus, it will be beneficial for the app design to have minimum friction to access recommended content, preferably on the home page of the app. Further, since longer conversational sessions drive more relevant content recommendations, we want to ensure that we trigger conversation-based recommendations only for sessions with more than a threshold number of messages. As previously discussed, we have already incorporated this design decision into our recommendation infrastructure. While conversation-based recommendations may provide better engagement, the most suitable algorithm will depend on the context of usage. Onboarding-based recommendations are ideal for “cold starting” the process of recommending content for new users and users that tend to use the app just for content but not for therapy or coaching [27]. Conversation-based recommendations, on the other hand, change adaptively with the topics that users discuss with their coaches. These can be used to immerse more deeply in the coaching journey through personalized homework and activities. Finally, one should be mindful of how demographics can play a role in how sensitive users are to different levels and types of personalization [28]. 

### Conclusions 

Recommendation systems can help scale and supplement digital mental health care with personalized content and self-care recommendations. We present and evaluate 2 knowledge-based recommenders in this study: 1 static algorithm utilizing user onboarding responses and 1 adaptive algorithm utilizing user conversations with their coach. Onboarding-based recommendations are best suited for delivering personalized recommendations to users when there are sparse or skewed content usage data sets on a platform. On the other hand, the conversation-based recommendation algorithm allows for dynamic recommendations based on additional information gathered during text-based coaching sessions spanning months, which is essential given the changing nature of mental health needs throughout treatment. The conversation-based algorithm had the highest completion rates across all recommendation methods and other sections of the Ginger app that deliver content. This algorithm also had a higher completion rate among users aged 35 years and up and male-identifying users. The proposed algorithms are but a step toward outcome-driven personalization in mental health. Future work will involve a robust causal evaluation of these algorithms using randomized control trials and consumer feedback–driven improvement of these algorithms to drive better clinical outcomes.

## Appendix

Multimedia Appendix 1 Message importance algorithm: development and evaluation.

## Abbreviations

IRB: institutional review board
MAR: majority agreement rate
RS: recommender system

## Data Availability

For this study, we used a data set of coaching conversations between mental health care patients and their care providers, which constitutes personal health information, and app analytics data from the Ginger mobile app, a subsidiary of Headspace Health. These data are not publicly available due to privacy reasons and to safeguard Headspace Health’s in-house analytics data.
