# Supplementary material for: Content Recommendation Systems in Web-Based Mental Health Care: Real-world Application and Formative Evaluation
Source: JMIR Form Res. 2023 Jan 19;7:e38831. doi: 10.2196/38831 (PMC9896352; doi:10.2196/38831)
Supplement: Multimedia Appendix 1 [file formative_v7i1e38831_app1.docx]

# APPENDIX

# Message Importance Algorithm

We developed a classifier to predict the importance of each message. This classifier allowed us to filter unimportant messages from important ones in a conversational session, thus minimizing the risk of the conversation-based recommendation model making irrelevant predictions when the messages in a context session are insignificant to the context of mental health. This message importance (also referred to as extractive summarization [1]) model using weak supervision [2]. In this machine learning paradigm, noisy, limited, or imprecise sources of data are used to provide a supervision signal for labeling large amounts of training data in a supervised setting. [3] We chose this paradigm because we would typically gather annotations by labeling this data to train a complex model through a completely supervised method. However, we need thousands of examples of messages to train a model for this complex problem of assigning importance to messages, which can be very time-consuming and have a large time and opportunity cost. A dataset of training data generated using a weakly supervised model can solve this problem.

### Generating training data using weak supervision

To generate a surrogate dataset to train message importance, we first built an algorithm (which we call the similarity matching algorithm) that was trained to map out similarities and differences between messages in the session transcript and sentences in the corresponding coach note of that session (after every conversational session, Ginger’s coaches write a note summarizing the session and member’s progress). Once trained, we then used this similarity matching algorithm to generate labels for 28,415 messages spanning 500 Ginger conversations. These labels assigned the labels **Important** or **Not Important** to messages in these conversations. The process of developing the similarity matching algorithm is described in detail below.

### Similarity matching algorithm

With the goal of generating a large surrogate dataset that could be used to train a message importance classifier, we built an algorithm to map out overlapping content between coach notes and conversation sessions. These were developed to serve as a proxy for annotation for extractive summarization, assuming that similarities between the coach notes and session transcripts give us an important signal about what messages are important.

We did this to bypass the problem of not having sufficient labeled data to train an extractive summarization model which could be very expensive given this is a complex higher order task that would need many samples (i.e., 10,000 + messages) labeled by a certified mental health coach or therapist . The semi-supervised approach was informed by prior work [2] on weakly-supervised training and Rapid Training Data Creation[3]. This method of creating a surrogate dataset to train a separate model also allows us to inject domain knowledge into AI.


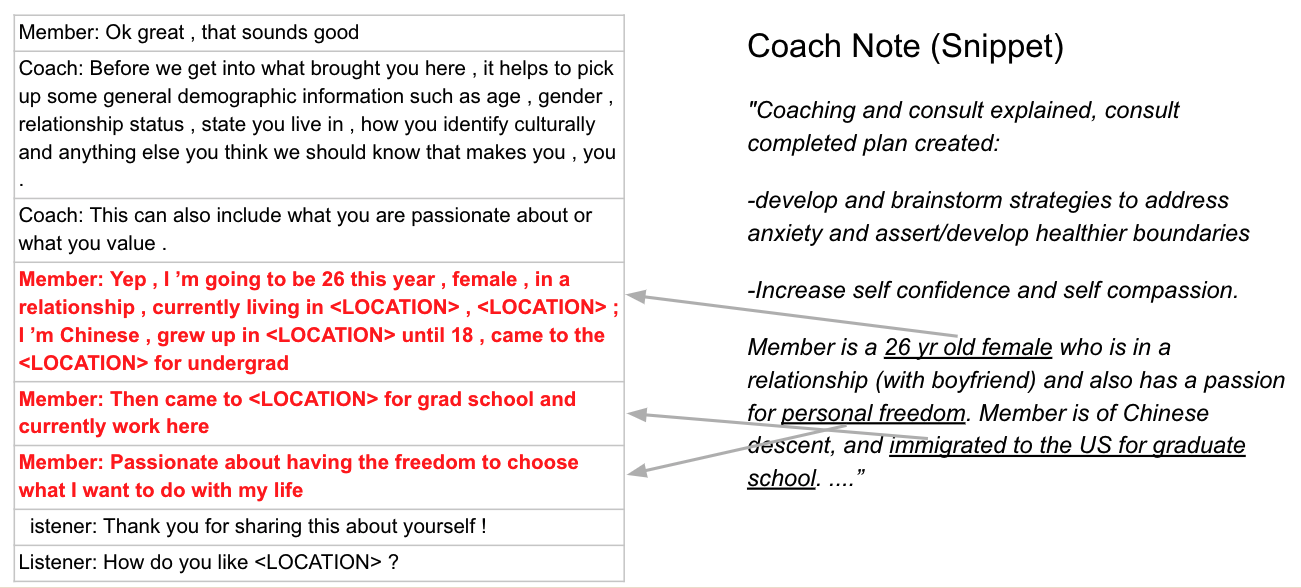


***Figure 6****. Mapping similarities between coach notes and conversational transcripts. Messages in red have high relevance to the coach note based on semantic similarity (explained via text underlined in the coach note).*

#### Dataset for training and validation

The data consisted of 12 conversations from the coach annotation for the Important Messages task, where the annotators had to identify if a message had overlapping content with its coach note (see figure 6 above). We split the training and validation in a ratio of 80:20.

#### Input features for the similarity matching algorithm/model

1. BM25 similarity between messages in the session and sentences in the coach summaries
2. The vector difference between the document embedding of message and the document embedding of a coach note
3. Cosine similarity score of document embeddings between message in the session and coach summaries
4. Length of a message
5. Presence of a question in message
6. Sender type that sent the message (coach or member)

#### Definitions

*BM25 similarity between messages in the session and sentences in the coach summaries*

1. We implemented the popular BM25 document retrieval algorithm to get similarity scores (weights) between ‘n’ sentences and *‘m’* messages in the session transcript which yielded an m x n similarity matrix.
2. To get a measure of the importance of a transcript message, we flattened this matrix by taking every column’s max yielding an array, which was used as the final feature for every message.

*The vector difference between the document embedding of messages and the embedding of a whole coach note*

1. The most important feature in this model, this vector captures the overall semantic similarities and differences between the message and the coach note.
2. It is simply a vector subtraction of the two embedding vectors.

*Similarity metrics using Sentence Bert embeddings between messages in the session and sentences in the coach summaries*

1. While the above metric gets the similarity of a message with the whole coach note, this metric gets the similarity of a message with individual sentences in the coach note, offering more granularity.
2. We computed sentence embeddings using the Sentence Bert library to get similarity scores (weights) between ‘n’ sentences and, *‘m’* ’messages in the session transcript which yielded an m x n similarity matrix.
3. To measure the importance of a transcript message, we flattened this matrix by taking every column’s max value yielding an array, which was used as the final feature for every message.

*Length of a transcript message, presence of a question in a transcript message, and sender type - coach or member:*

1. Very simply, the token length of a message, a boolean that confirms if a message has questions in it, and the sender-type of a conversational message.

We trained an XGBoost classifier to generate labels for downstream processing by an extractive summarization model.
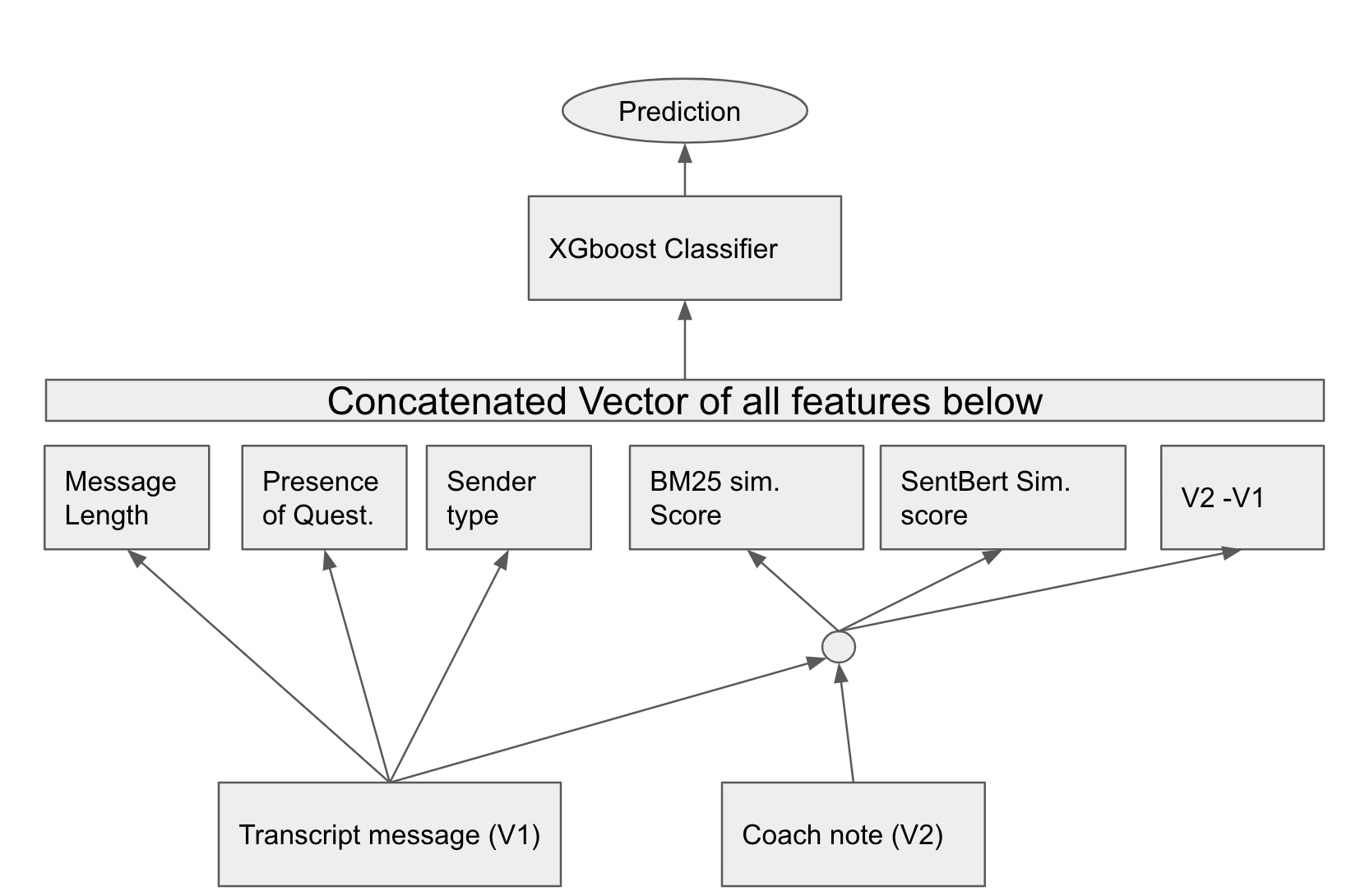


***Figure 7.*** *Model Architecture for the similarity algorithm. V1 and V2 are the embeddings of a message and the whole coach notes, respectively.*

For each conversation session, annotators had to identify which messages in a conversation transcript had **overlapping content** with the corresponding coach note. A message was labeled **Important** if its information/content could have been used to write some portion of the coach note or if it is a paraphrase or direct excerpt of some portion of the coach note. Overall we achieved a macro average F1 score of 0.74 and an overall model accuracy of 87%.

We ran inference on this model on an unlabeled pool of messages to get **Important/Not-important** labels generated for a dataset of 28,415 labeled messages.

### Building the Message Importance Classifier

Using the 28,415 messages we generated using weak supervision from the similarity matching algorithm, we trained a gradient boosted model (XGboost) model that could detect if a message in a session is important or not. This model’s input feature was the Universal Sentence Encoder embeddings of the messages computed using a pre-trained model from tensorflow hub [4]. We evaluated this algorithm on a holdout set of 12 conversations collected via coach annotations with annotator overlap. The balanced majority agreement rate (F1 score) on the holdout dataset over all the annotators is 0.863. We achieved a macro average F1 score of 0.74 with a balanced accuracy of 0.77 for this model on the holdout set.

References

1. Zhong, M., Liu, P., Chen, Y., Wang, D., Qiu, X. and Huang, X., 2020. Extractive summarization as text matching. arXiv preprint arXiv:2004.08795.

2[. Ratner, Alex, Varma, Paroma, Hancock, Braden, Re, Chris. Weak Supervision: A New Programming Paradigm for Machine Learning. SAIL Blog. Published March 10, 2019. Accessed April 7, 2022. http://ai.stanford.edu/blog/weak-supervision/](https://www.zotero.org/google-docs/?BJAtHU)

3[. Ratner A, Bach SH, Ehrenberg H, Fries J, Wu S, Ré C. Snorkel: Rapid Training Data Creation with Weak Supervision. *Proc VLDB Endow*. 2017;11(3):269-282. doi:10.14778/3157794.3157797](https://www.zotero.org/google-docs/?BJAtHU)

4[. TensorFlow Hub. Accessed April 10, 2022. https://tfhub.dev/google/universal-sentence-encoder/4](https://www.zotero.org/google-docs/?BJAtHU)
